# Supplementary material for: Hot spot prediction in protein-protein interactions by an ensemble system
Source: BMC Syst Biol. 2018 Dec 31;12(Suppl 9):132. doi: 10.1186/s12918-018-0665-8 (PMC6311905; doi:10.1186/s12918-018-0665-8)
Supplement: Supplementary file 2 — All relevant MATLAB code. A simple Matlab implement of our predictor. (ZIP 19,742 kb) [file 12918_2018_665_MOESM2_ESM.zip › Additional file 2/Read ASA/ASA website/dbMPIKT/4FZAA.html]

 NetSurfP - Protein Surface Accessibility and Secondary Structure Predictions

  

|  |  |  |
| --- | --- | --- |
|  |  | NetSurfP - Protein Surface Accessibility and Secondary Structure Predictions  Technical University of Denmark |

  


---

```
# For publication of results, please cite:
# A generic method for assignment of reliability scores applied to solvent accessibility predictions.
# Bent Petersen, Thomas Nordahl Petersen, Pernille Andersen, Morten Nielsen and Claus Lundegaard
# BMC Structural Biology 2009, 9:51 doi:10.1186/1472-6807-9-51
#
# Column 1: Class assignment - B for buried or E for Exposed - Threshold: 25% exposure, but not based on RSA
# Column 2: Amino acid
# Column 3: Sequence name
# Column 4: Amino acid number
# Column 5: Relative Surface Accessibility - RSA
# Column 6: Absolute Surface Accessibility
# Column 7: Z-fit score for RSA prediction
# Column 8: Probability for Alpha-Helix
# Column 9: Probability for Beta-strand
# Column 10: Probability for Coil
E G  4FZAA                  1    0.954  75.048  -1.072   0.003   0.003   0.994
E A  4FZAA                  2    0.726  80.027  -0.129   0.018   0.019   0.964
E M  4FZAA                  3    0.571 114.157  -1.060   0.018   0.019   0.964
E A  4FZAA                  4    0.453  49.888   0.037   0.016   0.005   0.979
E S  4FZAA                  5    0.426  49.927   0.784   0.016   0.005   0.979
B P  4FZAA                  6    0.120  16.971  -0.541   0.694   0.003   0.303
E A  4FZAA                  7    0.370  40.785  -0.183   0.923   0.002   0.076
E D  4FZAA                  8    0.506  72.886   0.864   0.923   0.002   0.076
B I  4FZAA                  9    0.067  12.377   0.959   0.970   0.001   0.030
B V  4FZAA                 10    0.029   4.488   0.710   0.970   0.001   0.030
E K  4FZAA                 11    0.432  88.862   1.736   0.970   0.001   0.030
B N  4FZAA                 12    0.262  38.298   1.410   0.970   0.001   0.030
B L  4FZAA                 13    0.031   5.658   0.549   0.970   0.001   0.030
B K  4FZAA                 14    0.250  51.322   0.822   0.970   0.001   0.030
E E  4FZAA                 15    0.504  88.031   0.805   0.970   0.001   0.030
B S  4FZAA                 16    0.114  13.384  -0.142   0.970   0.001   0.030
B M  4FZAA                 17    0.065  13.006   0.493   0.970   0.001   0.030
E A  4FZAA                 18    0.382  42.074   0.633   0.923   0.002   0.076
E V  4FZAA                 19    0.335  51.566   0.746   0.858   0.002   0.139
B L  4FZAA                 20    0.079  14.465  -0.018   0.782   0.003   0.216
E E  4FZAA                 21    0.362  63.224  -0.066   0.622   0.015   0.363
E K  4FZAA                 22    0.644 132.471   0.247   0.321   0.003   0.675
E Q  4FZAA                 23    0.454  81.031  -0.751   0.109   0.005   0.886
E D  4FZAA                 24    0.645  93.002  -0.117   0.053   0.005   0.942
E I  4FZAA                 25    0.378  70.004  -1.084   0.053   0.005   0.942
E S  4FZAA                 26    0.557  65.327  -1.087   0.109   0.005   0.886
E D  4FZAA                 27    0.608  87.627  -1.102   0.406   0.004   0.590
E K  4FZAA                 28    0.665 136.852   1.026   0.782   0.003   0.216
E K  4FZAA                 29    0.377  77.590   0.526   0.858   0.002   0.139
B A  4FZAA                 30    0.220  24.266   0.355   0.923   0.002   0.076
E E  4FZAA                 31    0.482  84.170   0.763   0.923   0.002   0.076
E K  4FZAA                 32    0.450  92.606   1.535   0.970   0.001   0.030
B A  4FZAA                 33    0.080   8.816   0.381   0.970   0.001   0.030
B T  4FZAA                 34    0.124  17.185   0.697   0.970   0.001   0.030
E E  4FZAA                 35    0.449  78.458   0.772   0.988   0.000   0.012
E E  4FZAA                 36    0.328  57.249   0.712   0.988   0.000   0.012
B V  4FZAA                 37    0.028   4.350   0.821   0.988   0.000   0.012
B S  4FZAA                 38    0.124  14.556   0.183   0.970   0.001   0.030
E K  4FZAA                 39    0.507 104.249   1.239   0.970   0.001   0.030
B N  4FZAA                 40    0.146  21.301   0.355   0.970   0.001   0.030
B L  4FZAA                 41    0.042   7.672   0.486   0.970   0.001   0.030
E V  4FZAA                 42    0.377  57.868   1.030   0.970   0.001   0.030
E A  4FZAA                 43    0.371  40.873   0.870   0.938   0.007   0.055
B M  4FZAA                 44    0.033   6.603   0.461   0.938   0.007   0.055
B K  4FZAA                 45    0.216  44.472   1.070   0.938   0.007   0.055
E E  4FZAA                 46    0.440  76.833   0.483   0.911   0.033   0.057
B I  4FZAA                 47    0.119  21.960   1.116   0.831   0.044   0.125
B L  4FZAA                 48    0.043   7.818   0.073   0.701   0.107   0.192
E Y  4FZAA                 49    0.289  61.781   0.306   0.307   0.165   0.527
E G  4FZAA                 50    0.420  33.054  -0.361   0.053   0.043   0.903
E T  4FZAA                 51    0.526  72.901  -0.430   0.018   0.019   0.964
E N  4FZAA                 52    0.672  98.352  -0.826   0.016   0.005   0.979
E E  4FZAA                 53    0.562  98.251  -1.064   0.053   0.005   0.942
E K  4FZAA                 54    0.643 132.327   0.110   0.058   0.017   0.925
E E  4FZAA                 55    0.450  78.580   0.005   0.058   0.017   0.925
E P  4FZAA                 56    0.471  66.821  -0.080   0.058   0.017   0.925
E Q  4FZAA                 57    0.348  62.099   0.665   0.176   0.004   0.820
E T  4FZAA                 58    0.490  67.991   0.242   0.923   0.002   0.076
E E  4FZAA                 59    0.658 114.970   0.429   0.970   0.001   0.030
B A  4FZAA                 60    0.218  24.013   1.124   0.970   0.001   0.030
B V  4FZAA                 61    0.050   7.716   0.939   0.988   0.000   0.012
E A  4FZAA                 62    0.333  36.664   1.064   0.970   0.001   0.030
E Q  4FZAA                 63    0.398  71.101   1.391   0.988   0.000   0.012
B L  4FZAA                 64    0.048   8.844   1.041   0.970   0.001   0.030
B A  4FZAA                 65    0.057   6.259   0.636   0.988   0.000   0.012
E Q  4FZAA                 66    0.350  62.456   1.189   0.970   0.001   0.030
E E  4FZAA                 67    0.290  50.646   1.158   0.970   0.001   0.030
B L  4FZAA                 68    0.058  10.620   0.742   0.970   0.001   0.030
B Y  4FZAA                 69    0.114  24.469   1.096   0.858   0.002   0.139
E N  4FZAA                 70    0.460  67.359   0.474   0.782   0.003   0.216
E S  4FZAA                 71    0.305  35.758   0.195   0.600   0.003   0.397
E G  4FZAA                 72    0.353  27.781  -0.632   0.321   0.003   0.675
B L  4FZAA                 73    0.050   9.137   1.217   0.858   0.002   0.139
B L  4FZAA                 74    0.041   7.599   0.720   0.975   0.003   0.022
B S  4FZAA                 75    0.213  24.952   0.975   0.975   0.003   0.022
B T  4FZAA                 76    0.174  24.120   0.551   0.970   0.001   0.030
B L  4FZAA                 77    0.028   5.108   0.602   0.970   0.001   0.030
B V  4FZAA                 78    0.055   8.392  -0.547   0.970   0.001   0.030
E A  4FZAA                 79    0.321  35.374   0.921   0.923   0.002   0.076
B D  4FZAA                 80    0.182  26.183   0.372   0.858   0.002   0.139
B L  4FZAA                 81    0.050   9.173   0.834   0.502   0.002   0.495
E Q  4FZAA                 82    0.455  81.299   0.468   0.321   0.003   0.675
E L  4FZAA                 83    0.526  96.256   1.169   0.246   0.004   0.750
B I  4FZAA                 84    0.093  17.113   0.676   0.053   0.005   0.942
E D  4FZAA                 85    0.667  96.057   0.563   0.003   0.003   0.994
B F  4FZAA                 86    0.186  37.250   0.022   0.109   0.005   0.886
E E  4FZAA                 87    0.669 116.857   0.822   0.246   0.004   0.750
E G  4FZAA                 88    0.249  19.573  -0.002   0.430   0.016   0.555
B K  4FZAA                 89    0.272  56.033   0.265   0.622   0.015   0.363
E K  4FZAA                 90    0.394  80.943   0.472   0.802   0.014   0.185
B D  4FZAA                 91    0.244  35.088   0.445   0.923   0.002   0.076
B V  4FZAA                 92    0.026   3.965   0.845   0.975   0.003   0.022
B A  4FZAA                 93    0.093  10.249   0.313   0.975   0.003   0.022
E Q  4FZAA                 94    0.278  49.687   1.561   0.992   0.000   0.007
B I  4FZAA                 95    0.031   5.754   0.623   0.992   0.000   0.007
B F  4FZAA                 96    0.048   9.614   0.774   0.988   0.000   0.012
B N  4FZAA                 97    0.098  14.289  -0.071   0.970   0.001   0.030
B N  4FZAA                 98    0.135  19.793   0.089   0.970   0.001   0.030
B I  4FZAA                 99    0.036   6.605  -0.029   0.938   0.007   0.055
B L  4FZAA                100    0.070  12.817  -0.092   0.938   0.007   0.055
E R  4FZAA                101    0.347  79.577   0.735   0.717   0.014   0.269
E R  4FZAA                102    0.289  66.181   0.875   0.561   0.047   0.393
E Q  4FZAA                103    0.347  61.974   0.863   0.184   0.043   0.773
E I  4FZAA                104    0.322  59.551  -0.443   0.115   0.016   0.868
E G  4FZAA                105    0.715  56.294  -1.096   0.016   0.005   0.979
E T  4FZAA                106    0.632  87.658  -1.627   0.016   0.005   0.979
E R  4FZAA                107    0.562 128.812  -0.097   0.016   0.005   0.979
B T  4FZAA                108    0.281  39.030   0.048   0.016   0.005   0.979
B P  4FZAA                109    0.072  10.174  -0.134   0.113   0.043   0.844
B T  4FZAA                110    0.099  13.731   0.397   0.453   0.248   0.299
B V  4FZAA                111    0.073  11.282  -0.023   0.701   0.107   0.192
E E  4FZAA                112    0.450  78.615   1.236   0.911   0.033   0.057
B Y  4FZAA                113    0.144  30.794   1.211   0.879   0.010   0.111
B I  4FZAA                114    0.032   5.902   0.388   0.879   0.010   0.111
B C  4FZAA                115    0.186  26.072  -0.361   0.802   0.014   0.185
E T  4FZAA                116    0.522  72.346   0.456   0.622   0.015   0.363
B Q  4FZAA                117    0.205  36.649   1.405   0.181   0.016   0.803
B Q  4FZAA                118    0.196  35.006   0.930   0.782   0.003   0.216
E N  4FZAA                119    0.546  79.949   1.026   0.858   0.002   0.139
B I  4FZAA                120    0.044   8.048   0.711   0.970   0.001   0.030
B L  4FZAA                121    0.025   4.632   1.291   0.970   0.001   0.030
B F  4FZAA                122    0.205  41.063   0.102   0.988   0.000   0.012
B M  4FZAA                123    0.161  32.296   0.648   0.970   0.001   0.030
B L  4FZAA                124    0.031   5.639   0.440   0.970   0.001   0.030
B L  4FZAA                125    0.068  12.469  -0.690   0.923   0.002   0.076
E K  4FZAA                126    0.408  83.946   0.957   0.782   0.003   0.216
B G  4FZAA                127    0.182  14.316  -1.321   0.406   0.004   0.590
B Y  4FZAA                128    0.118  25.302   0.418   0.176   0.004   0.820
E E  4FZAA                129    0.611 106.829   0.070   0.053   0.005   0.942
E S  4FZAA                130    0.302  35.359   0.340   0.058   0.017   0.925
E P  4FZAA                131    0.383  54.390  -0.539   0.257   0.016   0.727
E E  4FZAA                132    0.423  73.916  -0.748   0.339   0.016   0.645
B I  4FZAA                133    0.069  12.728  -0.570   0.622   0.015   0.363
B A  4FZAA                134    0.059   6.447  -1.487   0.660   0.049   0.291
B L  4FZAA                135    0.151  27.630  -0.780   0.717   0.014   0.269
B N  4FZAA                136    0.178  26.103  -0.439   0.717   0.014   0.269
B C  4FZAA                137    0.041   5.770  -0.536   0.694   0.003   0.303
B G  4FZAA                138    0.077   6.028  -1.227   0.694   0.003   0.303
B I  4FZAA                139    0.143  26.455  -0.447   0.858   0.002   0.139
B M  4FZAA                140    0.035   6.963   0.020   0.938   0.007   0.055
B L  4FZAA                141    0.017   3.058   1.466   0.938   0.007   0.055
B R  4FZAA                142    0.149  34.052   0.354   0.938   0.007   0.055
B E  4FZAA                143    0.101  17.680  -0.361   0.879   0.010   0.111
B C  4FZAA                144    0.025   3.524   0.518   0.802   0.014   0.185
B I  4FZAA                145    0.038   7.067   0.144   0.622   0.015   0.363
E R  4FZAA                146    0.390  89.287   0.964   0.339   0.016   0.645
B H  4FZAA                147    0.184  33.506   0.645   0.430   0.016   0.555
E E  4FZAA                148    0.435  75.925   0.674   0.622   0.015   0.363
E P  4FZAA                149    0.410  58.193   0.564   0.717   0.014   0.269
B L  4FZAA                150    0.046   8.514  -0.057   0.751   0.050   0.199
B A  4FZAA                151    0.089   9.830   0.499   0.701   0.107   0.192
E K  4FZAA                152    0.433  89.150   1.678   0.649   0.163   0.188
B I  4FZAA                153    0.146  27.028   0.585   0.578   0.229   0.194
B I  4FZAA                154    0.086  15.873   0.674   0.352   0.332   0.316
B L  4FZAA                155    0.078  14.190  -0.412   0.148   0.418   0.435
E W  4FZAA                156    0.392  94.228   0.326   0.064   0.216   0.721
E S  4FZAA                157    0.407  47.654   0.022   0.184   0.043   0.773
E E  4FZAA                158    0.754 131.759   0.090   0.600   0.003   0.397
E Q  4FZAA                159    0.255  45.489   0.909   0.858   0.002   0.139
B F  4FZAA                160    0.036   7.125   0.092   0.923   0.002   0.076
E Y  4FZAA                161    0.296  63.277   0.842   0.938   0.007   0.055
E D  4FZAA                162    0.414  59.614   1.573   0.938   0.007   0.055
B F  4FZAA                163    0.052  10.336   0.952   0.938   0.007   0.055
B F  4FZAA                164    0.050  10.035   0.614   0.879   0.010   0.111
E R  4FZAA                165    0.462 105.798   1.611   0.879   0.010   0.111
B Y  4FZAA                166    0.188  40.133   0.249   0.879   0.010   0.111
B V  4FZAA                167    0.055   8.484   0.193   0.751   0.050   0.199
E E  4FZAA                168    0.514  89.778  -0.522   0.455   0.046   0.498
B M  4FZAA                169    0.252  50.485  -0.084   0.181   0.016   0.803
E S  4FZAA                170    0.469  55.002  -0.811   0.115   0.016   0.868
B T  4FZAA                171    0.167  23.163  -0.434   0.268   0.043   0.689
B F  4FZAA                172    0.072  14.370   0.237   0.605   0.105   0.290
E D  4FZAA                173    0.266  38.331   0.146   0.725   0.163   0.112
B I  4FZAA                174    0.076  14.097   0.916   0.725   0.163   0.112
B A  4FZAA                175    0.027   2.997   0.565   0.779   0.100   0.120
B S  4FZAA                176    0.105  12.353  -0.117   0.911   0.033   0.057
E D  4FZAA                177    0.272  39.152  -0.088   0.938   0.007   0.055
B A  4FZAA                178    0.036   3.923  -0.871   0.923   0.002   0.076
B F  4FZAA                179    0.087  17.421   0.090   0.970   0.001   0.030
E A  4FZAA                180    0.410  45.171   0.871   0.970   0.001   0.030
B T  4FZAA                181    0.127  17.601   0.429   0.970   0.001   0.030
B F  4FZAA                182    0.037   7.366   0.151   0.988   0.000   0.012
E K  4FZAA                183    0.329  67.634   1.389   0.988   0.000   0.012
E D  4FZAA                184    0.281  40.492   0.101   0.970   0.001   0.030
B L  4FZAA                185    0.049   8.954   0.361   0.970   0.001   0.030
B L  4FZAA                186    0.061  11.132  -0.120   0.923   0.002   0.076
E T  4FZAA                187    0.345  47.851  -0.272   0.782   0.003   0.216
E R  4FZAA                188    0.370  84.661   0.558   0.502   0.002   0.495
B H  4FZAA                189    0.147  26.685   0.257   0.109   0.005   0.886
E K  4FZAA                190    0.408  83.967   0.634   0.782   0.003   0.216
E L  4FZAA                191    0.642 117.532   0.387   0.879   0.010   0.111
B L  4FZAA                192    0.101  18.475   0.421   0.879   0.010   0.111
B S  4FZAA                193    0.033   3.868   0.004   0.938   0.007   0.055
E A  4FZAA                194    0.285  31.418   0.516   0.970   0.001   0.030
E E  4FZAA                195    0.537  93.849   1.283   0.970   0.001   0.030
B F  4FZAA                196    0.107  21.395   0.502   0.970   0.001   0.030
B L  4FZAA                197    0.052   9.576   0.281   0.970   0.001   0.030
E E  4FZAA                198    0.436  76.187   0.945   0.923   0.002   0.076
E Q  4FZAA                199    0.542  96.873   1.366   0.858   0.002   0.139
B H  4FZAA                200    0.199  36.162   0.437   0.694   0.003   0.303
B Y  4FZAA                201    0.076  16.284   1.368   0.923   0.002   0.076
E D  4FZAA                202    0.563  81.071   1.385   0.970   0.001   0.030
E R  4FZAA                203    0.373  85.509   1.968   0.970   0.001   0.030
B F  4FZAA                204    0.027   5.459   0.737   0.988   0.000   0.012
B F  4FZAA                205    0.062  12.504   1.063   0.988   0.000   0.012
E S  4FZAA                206    0.528  61.870   1.317   0.988   0.000   0.012
E E  4FZAA                207    0.273  47.728   1.900   0.988   0.000   0.012
B Y  4FZAA                208    0.039   8.398   0.524   0.988   0.000   0.012
B E  4FZAA                209    0.260  45.370   1.407   0.970   0.001   0.030
E K  4FZAA                210    0.439  90.364   1.645   0.970   0.001   0.030
B L  4FZAA                211    0.058  10.565   0.040   0.923   0.002   0.076
B L  4FZAA                212    0.070  12.762   0.144   0.782   0.003   0.216
E H  4FZAA                213    0.622 113.087   0.144   0.502   0.002   0.495
E S  4FZAA                214    0.390  45.696  -0.078   0.109   0.005   0.886
E E  4FZAA                215    0.665 116.158  -0.890   0.109   0.005   0.886
B N  4FZAA                216    0.263  38.474  -1.001   0.181   0.016   0.803
B Y  4FZAA                217    0.195  41.586   0.232   0.660   0.049   0.291
B V  4FZAA                218    0.126  19.305   0.098   0.831   0.044   0.125
B T  4FZAA                219    0.192  26.644   0.390   0.831   0.044   0.125
B K  4FZAA                220    0.142  29.127   0.593   0.879   0.010   0.111
B R  4FZAA                221    0.231  52.853   0.288   0.879   0.010   0.111
E Q  4FZAA                222    0.306  54.687   0.116   0.802   0.014   0.185
B S  4FZAA                223    0.067   7.899  -0.828   0.858   0.002   0.139
B L  4FZAA                224    0.050   9.063   0.712   0.923   0.002   0.076
E K  4FZAA                225    0.434  89.253   1.778   0.970   0.001   0.030
B L  4FZAA                226    0.099  18.090   0.418   0.970   0.001   0.030
B L  4FZAA                227    0.047   8.551   0.500   0.970   0.001   0.030
B G  4FZAA                228    0.168  13.237  -0.932   0.970   0.001   0.030
E E  4FZAA                229    0.399  69.653   1.097   0.970   0.001   0.030
B L  4FZAA                230    0.092  16.864   0.693   0.938   0.007   0.055
B L  4FZAA                231    0.094  17.211   0.019   0.782   0.003   0.216
B L  4FZAA                232    0.235  42.992  -0.125   0.502   0.002   0.495
E D  4FZAA                233    0.362  52.193   0.475   0.321   0.003   0.675
E R  4FZAA                234    0.455 104.103   0.363   0.600   0.003   0.397
E H  4FZAA                235    0.319  58.081  -0.603   0.782   0.003   0.216
B N  4FZAA                236    0.132  19.339  -0.416   0.782   0.003   0.216
B F  4FZAA                237    0.213  42.709   0.522   0.923   0.002   0.076
E T  4FZAA                238    0.494  68.476   1.148   0.970   0.001   0.030
B I  4FZAA                239    0.081  15.022   0.218   0.970   0.001   0.030
B M  4FZAA                240    0.090  17.989   0.043   0.970   0.001   0.030
E T  4FZAA                241    0.356  49.308   0.319   0.923   0.002   0.076
E K  4FZAA                242    0.387  79.606   1.333   0.879   0.010   0.111
B Y  4FZAA                243    0.111  23.742   0.398   0.802   0.014   0.185
B I  4FZAA                244    0.115  21.331  -0.671   0.522   0.016   0.462
E S  4FZAA                245    0.443  51.966  -0.832   0.181   0.016   0.803
E K  4FZAA                246    0.373  76.644   0.629   0.053   0.005   0.942
E P  4FZAA                247    0.487  69.134  -0.166   0.782   0.003   0.216
E E  4FZAA                248    0.562  98.269   0.560   0.782   0.003   0.216
B N  4FZAA                249    0.136  19.940   0.343   0.923   0.002   0.076
B L  4FZAA                250    0.029   5.328   0.585   0.970   0.001   0.030
E K  4FZAA                251    0.333  68.395   1.324   0.970   0.001   0.030
B L  4FZAA                252    0.092  16.772   0.844   0.970   0.001   0.030
B M  4FZAA                253    0.028   5.623   0.253   0.970   0.001   0.030
B M  4FZAA                254    0.047   9.505   0.861   0.970   0.001   0.030
E N  4FZAA                255    0.351  51.328   0.984   0.923   0.002   0.076
B L  4FZAA                256    0.091  16.735   0.275   0.858   0.002   0.139
B L  4FZAA                257    0.072  13.165  -0.369   0.694   0.003   0.303
E R  4FZAA                258    0.441 101.058   0.394   0.321   0.003   0.675
E D  4FZAA                259    0.391  56.285  -0.550   0.257   0.016   0.727
E K  4FZAA                260    0.618 127.164   0.054   0.181   0.016   0.803
E S  4FZAA                261    0.367  42.954  -0.879   0.181   0.016   0.803
E R  4FZAA                262    0.413  94.508   0.662   0.191   0.086   0.723
E N  4FZAA                263    0.372  54.475   0.296   0.148   0.418   0.435
B I  4FZAA                264    0.065  12.006   0.701   0.169   0.612   0.219
B Q  4FZAA                265    0.179  32.041   0.412   0.172   0.690   0.138
B F  4FZAA                266    0.107  21.495   0.125   0.169   0.612   0.219
B E  4FZAA                267    0.271  47.274   0.062   0.169   0.612   0.219
B A  4FZAA                268    0.054   5.995  -0.748   0.159   0.516   0.325
B F  4FZAA                269    0.095  19.127   0.261   0.079   0.592   0.329
B H  4FZAA                270    0.228  41.400   0.040   0.087   0.683   0.230
B V  4FZAA                271    0.053   8.208  -0.274   0.018   0.846   0.136
B F  4FZAA                272    0.075  15.012   0.111   0.018   0.846   0.136
B K  4FZAA                273    0.165  34.002   0.801   0.011   0.918   0.071
B V  4FZAA                274    0.029   4.457   0.843   0.011   0.918   0.071
B F  4FZAA                275    0.048   9.634   0.893   0.011   0.918   0.071
B V  4FZAA                276    0.043   6.640   0.029   0.018   0.846   0.136
B A  4FZAA                277    0.108  11.869  -0.799   0.022   0.552   0.426
B N  4FZAA                278    0.200  29.280   1.179   0.004   0.138   0.858
E P  4FZAA                279    0.581  82.430  -0.143   0.005   0.045   0.951
E N  4FZAA                280    0.669  97.956  -0.163   0.005   0.015   0.979
E K  4FZAA                281    0.416  85.612   0.183   0.016   0.005   0.979
B T  4FZAA                282    0.222  30.819  -0.204   0.109   0.005   0.886
E Q  4FZAA                283    0.651 116.269   1.026   0.858   0.002   0.139
E P  4FZAA                284    0.416  59.101   1.506   0.858   0.002   0.139
B I  4FZAA                285    0.022   3.977   0.909   0.923   0.002   0.076
B L  4FZAA                286    0.200  36.693   1.157   0.970   0.001   0.030
E D  4FZAA                287    0.562  80.927   2.006   0.970   0.001   0.030
B I  4FZAA                288    0.067  12.358   0.928   0.970   0.001   0.030
B L  4FZAA                289    0.041   7.580   0.297   0.975   0.003   0.022
E L  4FZAA                290    0.422  77.323   1.299   0.938   0.007   0.055
E K  4FZAA                291    0.552 113.526   1.556   0.858   0.002   0.139
B N  4FZAA                292    0.167  24.390   0.021   0.406   0.004   0.590
B Q  4FZAA                293    0.195  34.773   0.873   0.923   0.002   0.076
E A  4FZAA                294    0.653  71.983   0.981   0.970   0.001   0.030
E K  4FZAA                295    0.299  61.484   2.091   0.970   0.001   0.030
B L  4FZAA                296    0.019   3.497   1.355   0.970   0.001   0.030
B I  4FZAA                297    0.145  26.751   1.134   0.988   0.000   0.012
E E  4FZAA                298    0.554  96.731   1.937   0.970   0.001   0.030
B F  4FZAA                299    0.082  16.357   0.927   0.970   0.001   0.030
B L  4FZAA                300    0.051   9.393   0.541   0.923   0.002   0.076
E S  4FZAA                301    0.603  70.613   1.174   0.858   0.002   0.139
E K  4FZAA                302    0.678 139.465   1.450   0.694   0.003   0.303
B F  4FZAA                303    0.054  10.838   1.033   0.321   0.003   0.675
E Q  4FZAA                304    0.550  98.301   0.450   0.109   0.005   0.886
E N  4FZAA                305    0.404  59.102  -0.348   0.053   0.005   0.942
E D  4FZAA                306    0.727 104.717  -1.112   0.016   0.005   0.979
E R  4FZAA                307    0.434  99.317  -0.169   0.053   0.005   0.942
E T  4FZAA                308    0.482  66.867  -1.051   0.058   0.017   0.925
E E  4FZAA                309    0.592 103.492  -0.558   0.115   0.016   0.868
E D  4FZAA                310    0.331  47.639  -0.601   0.181   0.016   0.803
E E  4FZAA                311    0.679 118.621   0.348   0.339   0.016   0.645
E Q  4FZAA                312    0.473  84.496   1.197   0.430   0.016   0.555
B F  4FZAA                313    0.076  15.213   0.286   0.622   0.015   0.363
E N  4FZAA                314    0.495  72.527   0.858   0.879   0.010   0.111
E D  4FZAA                315    0.591  85.105   0.698   0.923   0.002   0.076
B E  4FZAA                316    0.258  45.055   0.320   0.970   0.001   0.030
B K  4FZAA                317    0.172  35.401   0.695   0.970   0.001   0.030
E T  4FZAA                318    0.452  62.720   0.871   0.970   0.001   0.030
E Y  4FZAA                319    0.335  71.547   1.767   0.970   0.001   0.030
B L  4FZAA                320    0.047   8.551   0.800   0.970   0.001   0.030
B V  4FZAA                321    0.079  12.065   0.204   0.988   0.000   0.012
E K  4FZAA                322    0.524 107.849   1.709   0.970   0.001   0.030
E Q  4FZAA                323    0.270  48.311   1.439   0.970   0.001   0.030
B I  4FZAA                324    0.078  14.449   0.029   0.923   0.002   0.076
E R  4FZAA                325    0.537 123.019   1.249   0.858   0.002   0.139
E D  4FZAA                326    0.712 102.599   1.517   0.600   0.003   0.397
E L  4FZAA                327    0.242  44.255   0.025   0.246   0.004   0.750
E K  4FZAA                328    0.880 181.037   1.079   0.003   0.003   0.994

Explain the output.  Go back.
```

---
